# Lobe-Specific Heterogeneity in Asymmetric Dimethylarginine and Matrix Metalloproteinase Levels in a Rat Model of Obstructive Cholestasis

**DOI:** 10.1155/2014/327537

**Published:** 2014-06-12

**Authors:** Andrea Ferrigno, Giuseppina Palladini, Alberto Bianchi, Vittoria Rizzo, Laura G. Di Pasqua, Stefano Perlini, Plinio Richelmi, Mariapia Vairetti

**Affiliations:** ^1^Department of Internal Medicine and Therapeutics, IRCCS S. Matteo Foundation, University of Pavia, 27100 Pavia, Italy; ^2^Department of Molecular Medicine, IRCCS S. Matteo Foundation, University of Pavia, 27100 Pavia, Italy

## Abstract

We investigated the effects of obstructive cholestasis in different hepatic lobes by evaluating asymmetric dimethylarginine (ADMA) (a nitric oxide synthase inhibitor), protein methyltransferase (PRMT) and dimethylarginine dimethylaminohydrolase (DDAH) (enzymes involved, resp., in its synthesis and degradation), the cationic transporter (CAT), and metalloproteinase (MMP) activity. Sixteen male Wistar rats underwent a 3-day cholestasis by common bile duct ligation (BDL) or sham operation. Blood samples and hepatic biopsies from left lobe (LL), median lobe (ML), and right lobe (RL) were collected. Serum hepatic enzymes, tissue ADMA, DDAH activity, CAT-2 protein, mRNA expression of DDAH and PRMT, and MMP-2 and MMP-9 activity were monitored. Cholestasis was confirmed by altered serum hepatic enzymes. Higher levels of tissue ADMA were detected in RL and ML as compared with LL. PRMT mRNA expression and DDAH activity did not differ among the lobes after BDL. CAT-2 levels are higher in the RL and ML in the sham-operated group. Higher activity in MMP-2 and MMP-9 was found in RL. In conclusion, after cholestasis an increase in hepatic ADMA in RL and ML was detected as well as tissue MMP-2 and MMP-9 activation in RL, supporting the evidence of functional heterogeneity among the liver lobes also occurring in an obstructive cholestasis model.

## 1. Introduction

Cholestasis, an impairment in bile formation that occurs in a wide variety of human liver diseases [1], is characterized by retention of bile salts which is associated with enhanced generation of reactive oxygen/nitrogen species and oxidative stress [2]. Experimental and clinical studies have been mainly focused on the cellular alterations taking place in an individual lobe, whereas no data are available on the changes affecting all the hepatic lobes evaluated simultaneously. There is increasing evidence of the functional heterogeneity between the individual liver lobes that revealed a striking and yet unexplained inter- and intralobular variability of acute hepatic necrosis as shown from heterogeneous damage distribution within a single lobe or among different lobes. Several studies reported marked lobe variation in the extent and distribution of tissue injury during chemical carcinogenesis [3], acetaminophen hepatotoxicity [4], copper distribution [5] and cirrhosis [6], and ischemia/reperfusion (*I/R*) [7]. One possible explanation may be associated with the distribution of the liver vascular system: there is an incomplete mixing of blood coming from the gastrointestinal tract and spleen, leading to variation in the delivery of nutrients and toxins to the liver lobes. For example, venous portal blood draining the stomach and the spleen tends to be directed to the left side of the liver. Another possible mechanism that may play a central role in such hepatic variability is a different lobar gradient of gene expression profiles [8] as for acetaminophen hepatotoxicity, that is, a situation in which the variability of lobar damage has been correlated with different regional gene expression [9]. Lobe heterogeneity in matrix metalloproteinases (MMPs) activation, a large family of calcium-dependent-zinc-containing endopeptidases, has been recently found in* I/R* injury [7].

Recent studies reported that in an experimental BDL model serum changes in asymmetric dimethylarginine (ADMA), an endogenous inhibitor of nitric oxide synthase (NOS) enzyme, occur [10]. ADMA synthesis involves intracellular enzymes termed protein methyltransferase (PRMT) while its metabolic degradation occurs by dimethylarginine dimethylaminohydrolase (DDAH), an enzyme that is widely distributed in rats and humans, but, in particular, in the liver, kidney, and pancreas [11, 12]. Nijveldt et al. provide a detailed insight into the liver's handling of dimethylarginine, showing how it plays a crucial role in the metabolism of ADMA, with DDAH [13]: studies of gene silencing or deletion in rodents have led to the conclusion that plasma levels of ADMA are regulated by DDAH-1 isoform, whereas the predominant importance of DDAH-2 lies in preserving the endothelial function [14]. Membrane ADMA transport occurs by cationic amino-acid transporters (CATs): liver abundantly expresses CATs, especially CAT-2A and CAT-2B, suggesting a higher uptake of ADMA in this organ as compared with heart, lung, and kidney [15].

The aim of the present study was therefore to investigate whether obstructive cholestasis does separately affect the function of left, median, and right liver lobes via modulation of ADMA levels, DDAH activity, mRNA expression of PMRT and DDAH, and protein CAT levels. The concomitant MMPs activity was also detected in all the hepatic lobes to evaluate the early ECM remodeling, since the rat model of BDL is also used to study the hepatic fibrosis [16].

## 2. Material and Methods

### 2.1. Animals

The use of animals in this experimental study was approved by the National Institute for Research, and the animals were cared for according to its guidelines. Sixteen male Wistar rats (250–300 g, Harlan-Nossan, Italy) with free access to water and food were used.

### 2.2. Materials

All reagents were of the highest grade of purity available and were obtained from local suppliers.

### 2.3. Obstructive Cholestasis Procedure

**Figure 1:**
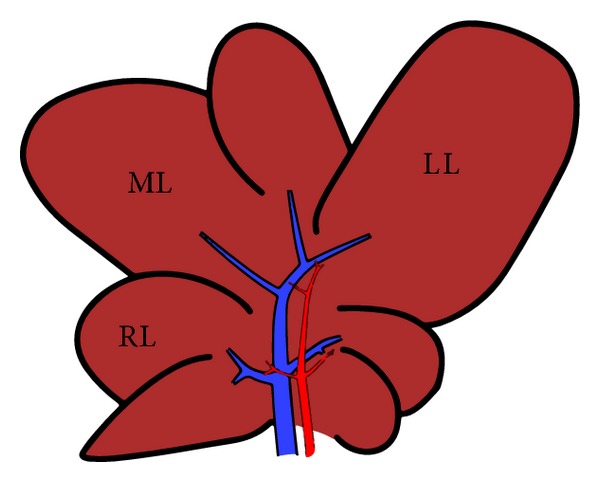
Graphic (schematic) representation of hepatic lobes in Sprague-Dawley rats: median lobe (ML), left lobe (LL), and right lobe (RL).

The abdomen was opened by a median incision in pentobarbital anesthetized rats (50 mg/kg) and the common bile duct was double-ligated and cut between the ligatures (BDL) (*n* = 8). Sham-operated control animals (*n* = 8) had similar manipulation but not bile duct ligation and were kept under anesthesia for an equal length of time. After 72 hours, blood samples were collected and immediately centrifuged to isolate serum. Hepatic biopsies from left lobe (LL), median lobe (ML), and right lobe (RL) (Figure 1) were collected and snap-frozen in liquid nitrogen.

### 2.4. Assays

Liver injury was assessed by serum levels of alanine transaminase (ALT), aspartate transaminase (AST), alkaline phosphatase (AP), and total and direct bilirubin by an automated Hitachi 747 analyser (Roche/Hitachi, Indianapolis, IN, USA). ADMA levels in plasma and tissue were evaluated by direct ELISA kit according to the manufacturing procedure (Immundiagnostik AG, Germany).

DDAH activity was evaluated using the method proposed by Tain and Baylis [17]. Tissue samples were homogenized in cold phosphate buffer 100 mM, pH 6.5; urease (100 U/mL) was added and samples were incubated at 37°C for 15 minutes; ADMA 1 mM in phosphate buffer was added (final ADMA concentration: 0.8 mM) and samples were incubated at 37°C for 60 minutes; the reaction was stopped by mixing 1 : 1 with 4% sulfosalicylic acid and samples were centrifuged for 10′ at 3000 g. Finally, the supernatants were assayed for citrulline as follows: Solution A (diacetyl monoxime 80 mM, thiosemicarbazide 2 mM) and Solution B (H_2_PO_4_ 3 M, H_2_SO_4_ 6 M, NH_4_Fe(SO_4_)_2_ 1.75 mM) were prepared, mixed 1 : 3, and added 1 : 1 to the samples. Samples were incubated at 60°C for 110 min and read spectrophotometrically at 528 nm against citrulline standards.

CAT-2 protein expression was evaluated by Rat CAT-2 ELISA kit (Cusabio, Wuhan University Science Park, Wuhan, China).

The extent of lipid peroxidation in terms of thiobarbituric acid reactive substances (TBARS) formation was measured according to the method of Esterbauer and Cheeseman [18]. TBARS concentrations were calculated using malondialdehyde (MDA) as standard.

### 2.5. Quantitative Real-Time PCR Analysis of Liver

DDAH-1 and PRMT mRNA were analysed by a real-time polymerase chain reaction (RT-PCR): total RNA was isolated from the liver samples with Trizol reagent according to the method of Chomczynski and Mackey [19]. RNA was quantified by measuring the absorbance at 260/280 nm. cDNA was generated using the iScript cDNA Synthesis kit (BIO-RAD) according to the supplier's instructions. Gene expression was analyzed using the SSO Advanced Sybr Green Supermix (BIO-RAD). In regard to housekeeping, ubiquitin C (UBC) gene and glyceraldehyde 3-phosphate dehydrogenase (GAPDH) gene were used (Table 1). DDAH-1, PRMT, UBC, and GAPDH gene amplification efficiency was 92,8%, 93,5%, 98,6%, and 97,4%, respectively, in a cDNA concentration range of 10–0,1 ng/*μ*L. The expression of the housekeeping gene remained constant in all the experimental groups considered. The amplification was performed through two-step cycling (95–60°C) for 40 cycles in a CFX Connect RT-PCR Detection System (BIO-RAD) following the supplier's instructions. All samples were assayed in triplicate. Gene expression was calculated using the ΔCt method. Comparison between groups was calculated using the ΔΔCt method.

### 2.6. Tissue Sources for MMPs Analysis

After sacrifice hepatic lobes were quickly excised and placed in cold (4°C) buffer (30 mM Histidine, 250 mM sucrose, 2 mM EDTA, pH 7.2) to remove blood. Liver was weighed and subsequently cut, frozen in liquid nitrogen, and stored at −80°C until use.

### 2.7. Hepatic MMPs Extraction and Zymography

Hepatic MMPs were extracted by homogenisation (IKA-ULTRA TURRAX T10) of frozen liver tissue, in an ice-cold extraction buffer (1 : 10 wt/vol) containing 1% Triton X-100, 500 mmol/L Tris-HCl, 200 mmol/L NaCl, and 10 mmol/L CaCl_2_, pH 7.6 [16]. The homogenate was then centrifuged (30 min at 12.000 rpm at 4°C) and the protein concentration of the supernatant was measured with the colorimetric Lowry method [20]. Samples were stored at −20°C before use. In order to detect MMPs activity present in the samples, the homogenate protein content was normalized by a final concentration of 400 *μ*g/mL in sample loading buffer (0.25 M Tris-HCl, 4% sucrose w/v, 10% SDS w/v, and 0.1% bromphenol blue w/v, pH 6.8). After dilution the samples were loaded onto electrophoretic gels (SDS-PAGE) containing 1 mg/mL of gelatin under nonreducing conditions [21] followed by zymography as described previously [22]. The zymograms were analyzed by densitometer (GS 710 Densitomer BIORAD, Hercules, CA, USA) and data were expressed as optical density (OD), reported to 1 mg/mL protein content.

### 2.8. Statistical Analysis

Results are expressed as mean ± standard error. Comparisons between groups were performed by unpaired* t*-test. When data distribution was not normal according to the Kolgonorov-Smrna test, Mann-Whitney test was used. Biochemical parameters were also analyzed by one-way ANOVA and where necessary by Kruskall-Wallis Test.

All statistical procedures were performed using the MedCalc statistical software package (11.6.0.0 version). A value of *P* < 0.05 was considered significant.

## 3. Results

### 3.1. Liver Injury

We used an established in vivo model of obstructive extrahepatic cholestasis which was confirmed by increase in serum enzymes: AST, ALT, and AP were higher in animals submitted to BDL as compared with the sham-operated group (control) (Table 2). Total and direct bilirubin concentrations were marked increased after BDL as compared with sham-operated rats (Table 2).

### 3.2. Tissue and Serum ADMA Levels

**Figure 2:**
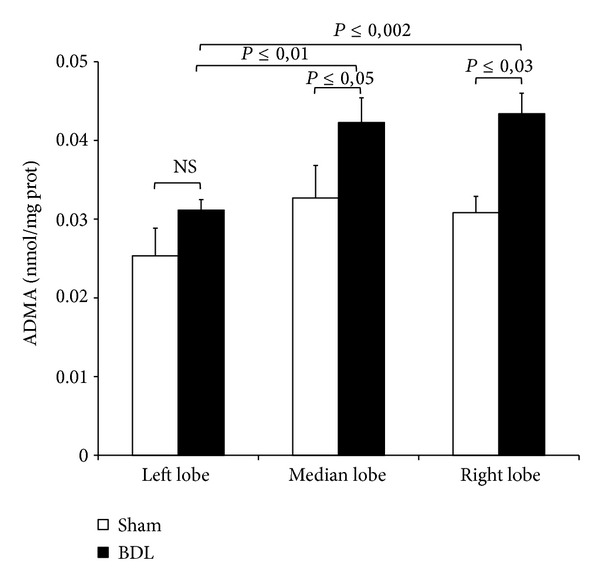
Hepatic levels of ADMA after 3-day BDL. Livers were submitted to BDL and left lobe (LL), median lobe (ML), and right lobe (RL) were collected. Sham-operated control animals had similar manipulation without bile duct occlusion. The results are reported as the mean ± S.E. of 8 different experiments.

The hepatic ADMA was comparable among lobes of sham-operated rats, while after 3-day BDL, tissue ADMA levels were significantly higher in RL and ML as compared with LL (Figure 2).

No changes in serum ADMA after BDL were detected as compared with sham-operated group (0,88 ± 0,06 versus 0,87 ± 0,02 nmol/mL, *P*≤ 0,49, resp.)

### 3.3. DDAH Expression and Activity

**Figure 3:**
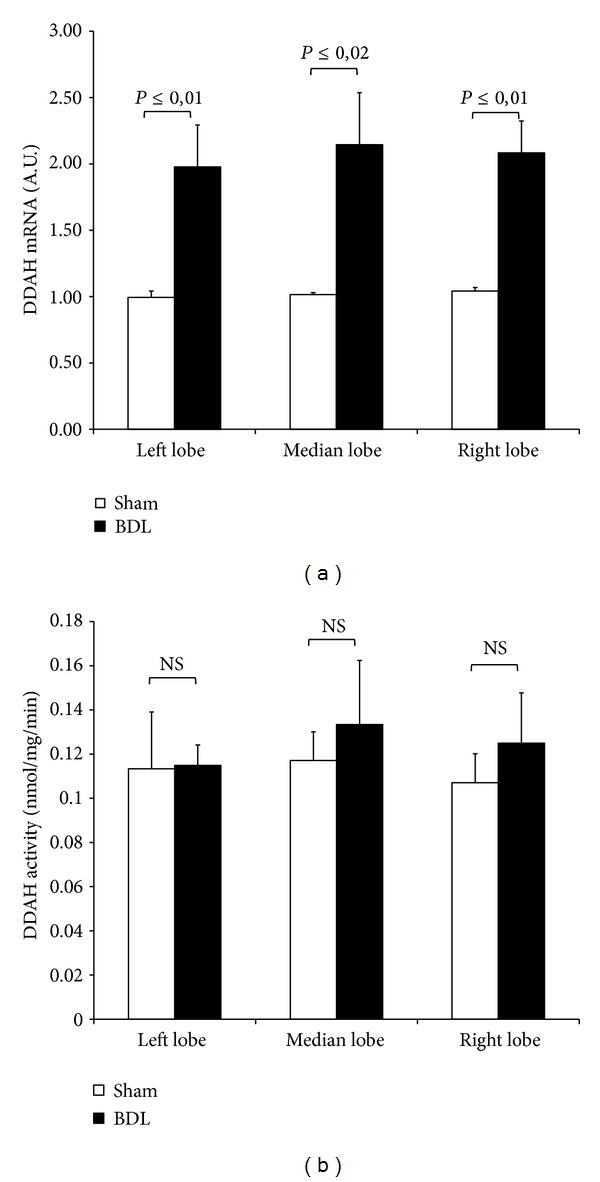
Tissue expression and activity of DDAH after 3-day BDL. Livers were submitted to BDL and left lobe (LL), median lobe (ML), and right lobe (RL) were collected. Sham-operated control animals had similar manipulation without bile duct occlusion. The results are reported as the mean ± S.E. of 8 different experiments.

**Figure 4:**
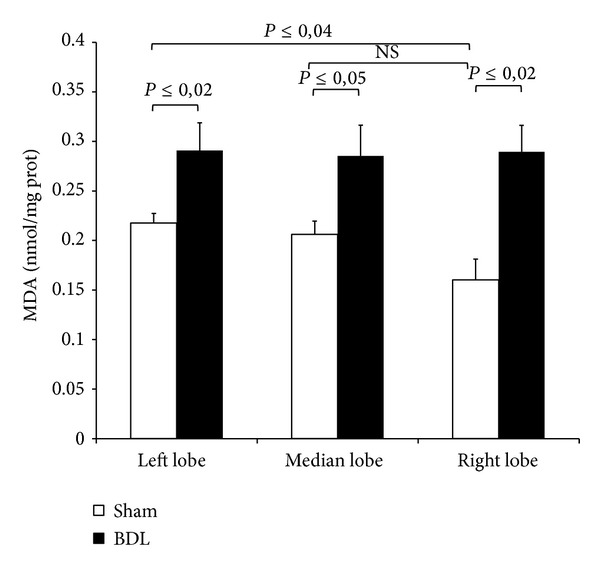
Hepatic levels of MDA after 3-day BDL. Livers were submitted to BDL and left lobe (LL), median lobe (ML), and right lobe (RL) were collected. Sham-operated control animals had similar manipulation without bile duct occlusion. The results are reported as the mean ± S.E. of 8 different experiments.

As reported in Figure 3(a), tissue DDAH-1 expression significantly increases in all tree lobes after 72-hour BDL as compared with the lobes, respectively, obtained from sham-operated group. No increase in DDAH activity was observed in RL, ML, and LL (Figure 3(b)). An explanation could be obtained by comparing the MDA levels detected in all tree lobes after BDL (Figure 4): higher oxidative stress was found as compared with the sham-operated group and this result is probably associated with a decrease in DDAH enzyme activity particularly susceptible to free radicals [23].

No difference in both DDAH expression and activity among the lobes was found in sham-operated group (Figure 3).

Of note, the analysis of MDA levels in sham-operated group confirms our previous published data in which RL showed lower levels of MDA as compared with LL [7].

### 3.4. Protein Methyltransferase (PRMT) Expression

**Figure 5:**
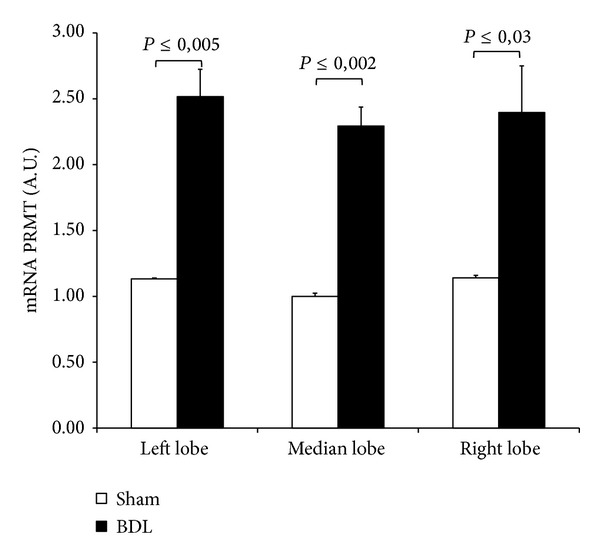
Tissue expression of PRMT after 3-day BDL. Livers were submitted to BDL and left lobe (LL), median lobe (ML), and right lobe (RL) were collected. Sham-operated control animals had similar manipulation without bile duct occlusion. The results are reported as the mean ± S.E. of 8 different experiments.

For evaluating the enzyme involved in the ADMA synthesis, the PRMT expression was detected after 72-hour BDL: significantly higher expression was found in all liver lobes when compared with the respective lobes from sham-operated group (Figure 5). No difference in PRMT expression among the lobes was found in sham-operated group or in BDL group (Figure 5).

### 3.5. Cationic Amino-Acid Transporters-2 (CAT-2) Protein

**Figure 6:**
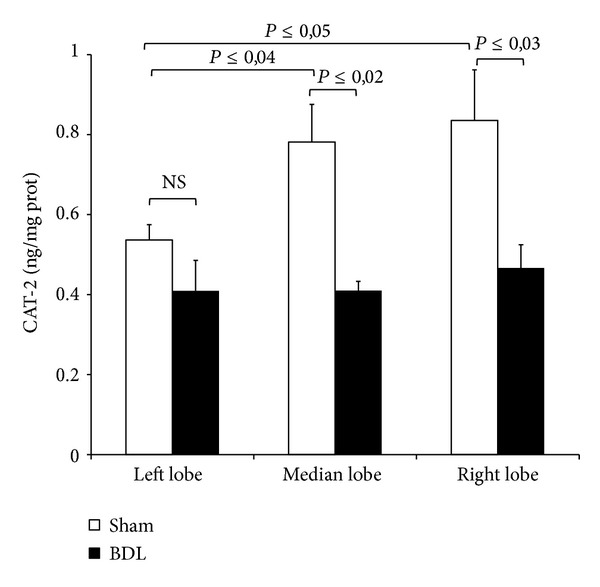
Hepatic CAT-2 protein after 3-day BDL. Livers were submitted to BDL and left lobe (LL), median lobe (ML), and right lobe (RL) were collected. Sham-operated control animals had similar manipulation without bile duct occlusion. The results are reported as the mean ± S.E. of 8 different experiments.

ADMA interferes with NO synthesis by competing with arginine and symmetric dimethylarginine (SDMA); the latter is not biologically active, for cellular transport across CAT. In sham-operated animals, higher levels of CAT-2 were found in RL and ML lobes as compared with LL (Figure 6). The isoform CAT-2, particularly expressed in the liver, significantly decreases in RL and ML after BDL as compared with the respective sham-operated group (Figure 6).

### 3.6. Gelatinolytic Activity

**Figure 7:**
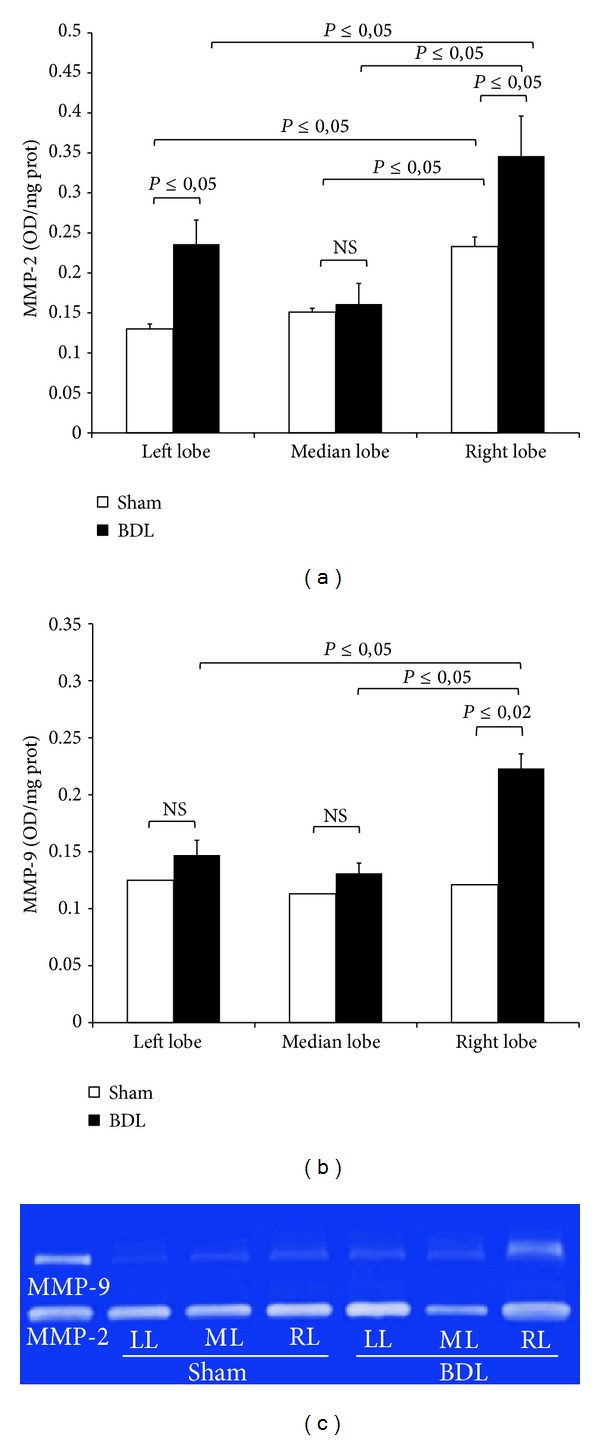
Bar graphs of hepatic MMP-2 (a) and MMP-9 (b) activity and gelatin zymography (c) after 3-day BDL. Livers were submitted to BDL and left lobe (LL), median lobe (ML), and right lobe (RL) were collected. Sham-operated control animals had similar manipulation without bile duct occlusion. Gelatinase activity of MMP-2 and MMP-9 is expressed as optical density (OD) for mm^2^, reported to 1 mg/mL protein content. Data are shown as mean values ± ES. (c) Representative gelatin zymography in homogenized liver tissue. Lanes 1: purified MMP-2 and MMP-9 proenzyme; lanes 2, 3, and 4: tissue samples of left (L), median (M), and right (R) sham-operated rats; lanes 5, 6, and 7: tissue samples of left (L), median (M), and right (R) BDL rats.

Gelatinase-A (MMP-2) and gelatinase-B (MMP-9) activities were evaluated in order to investigate the degree of MMP-induced hepatic extracellular matrix degradation after BDL and its variability among the different lobes. Both BDL and sham-operated groups showed detectable MMP-2 and MMP-9 activities. After 72 hours activities of MMP-2 increased significantly in BDL left and right lobes, but not in median lobe, as compared with the respective sham-operated group (Figure 7(a)). Interestingly, the RL obtained from sham-operated or BDL group showed higher levels of MMP-2 as compared with the respective ML and LL.

For MMP-9 significant differences were noted between BDL and sham-operated groups only in right lobe (Figure 7(b)). After BDL, the MMP-9 activity of RL was higher when compared with ML and LL.

## 4. Discussion

These results support previous data on the lobar functional heterogeneity of the liver: we found that, under the reported BLD conditions, the right and median lobes exhibit an increase in tissue ADMA concomitant with a marked CAT-2 decrease. After 3-day BDL, an activation of MMP-2 and MMP-9 occurs mainly in RL.

### 4.1. Lobe ADMA Heterogeneity after BDL

Recent studies reported an increase in serum ADMA levels evaluated 2 weeks after BDL [10, 24]; in the present work we evaluated what happened in a period close to the induction of damage. No increase in serum ADMA levels was found and it is reasonably connected with the period of occlusion: before obtaining an increase in serum, a tissue increase of ADMA occurs in the days immediately after BDL as reported in this study. In addition, to the best of our knowledge, this is the first evidence showing that ADMA is significantly higher in the right and median lobes as compared with the left lobe. In Wistar rats, the right and the caudate lobes represent a functional unit that differs from the middle and the left lobes in terms of protein synthesis [25]. On the other hand, it is also known that each lobe is organized into 3-dimensional vascular unit [26]. Moreover, while the left lobe has only one primary portal branch, the median lobe appears to have two portal branches [27]. The published data support the present finding on the hepatic lobe functionality in response to different pathological situation such as obstructive cholestasis.

To explain the hepatic ADMA increase in RL and ML, we evaluated the lobe heterogeneity of PRMT expression and no difference was detected among the lobes after BDL. The increase in DDAH mRNA expression after BDL was not found in the DDAH activity in RL, ML, and LL: as DDAH is highly oxidative sensitive enzyme, oxidative stress that occurs after BDL may inhibit DDAH activity as previously reported [23]. The heterogeneity of tissue ADMA concentration found after BDL is probably associated with the CAT-2 levels: these transporters were higher in the RL and ML in the sham-operated controls as compared with the LL; after BDL, a CAT-2 decrease was particularly evident in RL and ML when compared with the respective sham-operated groups. Previous papers reported that in obstructive cholestasis a downregulation of cationic transporter and its impairment occurs in rat liver already after 3-day BDL [28]. A decrease in CAT-2 transporters associated with an increase in tissue ADMA was also described in 2-week BDL [10]. CAT-2 is involved in both the cellular release and uptake of ADMA and the limitation in ADMA exchange could be the mechanism responsible for tissue ADMA concentration [29]. Furthermore, in humans the hepatic expression of the cationic drug uptake transporters in Caucasians is significantly affected by cholestasis [30].

The mechanism involved in this interlobe variation is largely unknown, although factors such as portal streamlining of blood to the liver [27] and differences in the metabolic capacity of each lobe have been proposed to explain the heterogeneous liver lobe response [31]. A variation in biliary drainage of the liver lobes could be also considered as specific event connected with the hepatic lobe heterogeneity [32].

### 4.2. Lobe MMPs Heterogeneity after BDL

Our results show marked alteration in gelatinases activity after BDL and show that this increase takes place in the first days after BDL mainly in the right lobe. The rat model of BDL is also used to study the hepatic fibrosis, a condition that results in loss of normal liver function due to changes of extracellular matrix (ECM) component [16]. It is important to emphasize that fibrosis progression is dynamic and constitutes both formation and degradation of ECM, which becomes imbalanced during liver disease [33–35]. During this remodeling process endopeptidases such as MMP-2 and MMP-9 are upregulated and are able to degrade excessive ECM.

Recent works support the crucial role of the analysis of the MMPs activation not only after 1-2 weeks after BDL but also after few days of occlusion [36, 37]. We confirm in rats the previous results in mice because we also observed an increase in MMP-2 and MMP-9 that in our conditions occurs significantly in RL than in ML and LL.

We previously reported a lobe-specific heterogeneity in MMP activation observed in a model of partial* I/R* injury: higher levels of MMP-2 and MMP-9 were detected also in nonischemic lobe (RL); in addition the MMP content was higher in RL of sham-operated group as compared with LL [7]. The MMP changes found in RL after* I/R* were oxidative-stress-independent and in the present work, using a BDL model, again this event appears to be oxidative-stress-independent. Based on this data it can be assumed that these markers reflect the early dynamics of fibrogenesis which would seem to start from the right lobe. Thus, they could be related to disease activity and may potentially also carry prognostic information.

The lobular heterogeneity seen in this cholestatic rat model was also found in other animal models of liver disease such as acetaminophen hepatotoxicity [9], cirrhosis [6], and* I/R* [7]. Although no data on lobular heterogeneity in human cholestatic liver diseases were reported, hepatic lobar differences have been described in subjects [38–41]. Of note, the human liver heterogeneity was documented in the progression of chronic disease: a proliferation of fibrosis was slower in left lobe than in the right [38].

In conclusion, these data indicate that different events occur in the different hepatic lobes after BDL injury. We reported, for the first time, that tissue ADMA increases in RL and ML with a CAT-2-dependent mechanism; this event is concomitant with a parallel MMP upregulation mainly in RL. In addition, the previously described lobe-specific heterogeneity has been confirmed and supported by the present study. Such elucidation of the hepatic heterogeneity may be useful in several clinical situations and it could help provide rational strategies for the treatment of cholestatic liver disorders.

## Figures and Tables

**Table 1:** List of forward and reverse primers used in experiments.

Gene	Sequence
Rat DDAH-1	Forward CAA CGA GGT CCT GAG ATC TTG GC
	Reverse GCA TCA GTA GAT GGT CCT TGA GC

Rat PRMT	Forward TGC TGC ACG CTC GTG ACA AGT
	Reverse TCC ACC ACG TCC ACC AGG GG

Rat UBC	Forward CAC CAA GAA CGT CAA ACA GGA A
	Reverse AAG ACA CCT CCC CAT CAA ACC

Rat GAPDH	Forward AAC CTG CCA AGT ATG ATG AC
	Reverse GGA GTT GCT GTT GAA GTC GTC A

**Table 2:** Serum enzymes and total and direct bilirubin levels in 3-day BDL and sham-operated rats.

	Sham	BDL
AST (mU/mL)	87,2 ± 8,3	647,7 ± 105*
ALT (mU/mL)	35,1 ± 2,5	308,3 ± 45*
Alcaline phosphatase (mU/mL)	264,5 ± 15	333,8 ± 33*
Total bilirubin (mg/dL)	0,08 ± 0,02	8,8 ± 0,13*
Direct bilirubin (mg/dL)	0,05 ± 0,01	7,04 ± 0,1*

*P < 0,05. These are the mean results of 8 different experiments ± S.E.

## Abbreviations

ADMA: Asymmetric arginine
PRMT: Protein methyltransferase
DDAH: Dimethylarginine dimethylaminohydrolase
CAT: Cationic transporters
MMP: Matrix metalloproteinase
BDL: Bile duct ligation
MDA: Malondialdehyde.

## References

[1] Miyoshi H, Rust C, Roberts PJ, Burgart LJ, Gores GJ (1999). Hepatocyte apoptosis after bile duct ligation in the mouse involves Fas. *Gastroenterology*.

[2] Portincasa P, Grattagliano I, Testini M (2007). Parallel intestinal and liver injury during early cholestasis in the rat: modulation by bile salts and antioxidants. *Free Radical Biology and Medicine*.

[3] Richardson FC, Boucheron JA, Dyroff MC, Popp JA, Swenberg JA (1986). Biochemical and morphologic studies of heterogeneous lobe responses in hepatocarcinogenesis. *Carcinogenesis*.

[4] Irwin RD, Parker JS, Lobenhofer EK (2005). Transcriptional profiling of the left and median liver lobes of male F344/N rats following exposure to acetaminophen. *Toxicologic Pathology*.

[5] Faa G, Nurchi V, Demelia L (1995). Uneven hepatic copper distribution in Wilson’s disease. *Journal of Hepatology*.

[6] Regev A, Berho M, Jeffers LJ (2002). Sampling error and intraobserver variation in liver biopsy in patients with chronic HCV infection. *American Journal of Gastroenterology*.

[7] Palladini G, Ferrigno A, Rizzo V (2012). Lobe-specific heterogeneity and matrix metalloproteinase activation after ischemia/reperfusion injury in rat livers. *Toxicologic Pathology*.

[8] Malarkey DE, Johnson K, Ryan L, Boorman G, Maronpot RR (2005). New insights into functional aspects of liver morphology. *Toxicologic Pathology*.

[9] Ruepp SU, Tonge RP, Shaw J, Wallis N, Pognan F (2002). Genomics and proteomics analysis of acetaminophen toxicity in mouse liver. *Toxicological Sciences*.

[10] Chang K, Lin I, Sheen J (2013). Sex differences of oxidative stress to cholestatic liver and kidney injury in young rats. *Pediatrics and Neonatology*.

[11] Kimoto M, Whitley GJ, Tsuji H, Ogawa T (1995). Detection of N(G),N(G)-dimethylarginine dimethylaminohydrolase in human tissues using a monoclonal antibody. *Journal of Biochemistry*.

[12] Kimoto M, Tsuji H, Ogawa T, Sasaoka K (1993). Detection of N(G),N(G)-dimethylarginine dimethylaminohydrolase in the nitric oxide-generating systems of rats using monoclonal antibody. *Archives of Biochemistry and Biophysics*.

[13] Nijveldt RJ, Teerlink T, Siroen MPC, Van Lambalgen AA, Rauwerda JA, Van Leeuwen PAM (2003). The liver is an important organ in the metabolism of asymmetrical dimethylarginine (ADMA). *Clinical Nutrition*.

[14] Palm F, Onozato ML, Luo Z, Wilcox CS (2007). Dimethylarginine dimethylaminohydrolase (DDAH): expression, regulation, and function in the cardiovascular and renal systems. *American Journal of Physiology-Heart and Circulatory Physiology*.

[15] Hattori Y, Kasai K, Gross SS (1999). Cationic amino acid transporter gene expression in cultured vascular smooth muscle cells and in rats. *American Journal of Physiology-Heart and Circulatory Physiology*.

[16] Kossakowska AE, Edwards DR, Lee SS (1998). Altered balance between matrix metalloproteinases and their inhibitors in experimental biliary fibrosis. *American Journal of Pathology*.

[17] Tain Y-L, Baylis C (2007). Determination of dimethylarginine dimethylaminohydrolase activity in the kidney. *Kidney International*.

[18] Esterbauer H, Cheeseman KH (1990). Determination of aldehydic lipid peroxidation products: malonaldehyde and 4-hydroxynonenal. *Methods in Enzymology*.

[19] Chomczynski P, Mackey K (1995). Substitution of chloroform by bromo-chloropropane in the single-step method of RNA isolation. *Analytical Biochemistry*.

[20] Lowry OH, Rosebrough NJ, Farr AL, Randall RJ (1951). Protein measurement with the Folin phenol reagent. *The Journal of Biological Chemistry*.

[21] Kleiner DE, Stetler-Stevenson WG (1994). Quantitative zymography: detection of picogram quantities of gelatinases. *Analytical Biochemistry*.

[22] Tozzi R, Palladini G, Fallarini S (2007). Matrix metalloprotease activity is enhanced in the compensated but not in the decompensated phase of pressure overload hypertrophy. *American Journal of Hypertension*.

[23] Tain Y, Kao Y, Hsieh C (2010). Melatonin blocks oxidative stress-induced increased asymmetric dimethylarginine. *Free Radical Biology and Medicine*.

[24] Yang Y, Lee T, Huang Y (2012). Asymmetric dimethylarginine (ADMA) determines the improvement of hepatic endothelial dysfunction by vitamin E in cirrhotic rats. *Liver International*.

[25] Garcia-Moreno L, Vallejo G, Arias JL, Aller M-A, Lorente L, Arias J (1994). Behaviour of nucleolar organizer regions in the different Wistar rat liver lobes. *Laboratory Animals*.

[26] Teutsch HF, Schuerfeld D, Groezinger E (1999). Three-dimensional reconstruction of parenchymal units in the liver of the rat. *Hepatology*.

[27] Duchen LW (1961). The effects of deprivation of portal blood on the liver and its influence on carbon tetrachloride liver injury in the rat. *British Journal of Experimental Pathology*.

[28] Denk GU, Soroka CJ, Mennone A, Koepsell H, Beuers U, Boyer JL (2004). Down-regulation of the organic cation transporter 1 of rat liver in obstructive cholestasis. *Hepatology*.

[29] Teerlink T, Luo Z, Palm F, Wilcox CS (2009). Cellular ADMA: regulation and action. *Pharmacological Research*.

[30] Nies AT, Koepsell H, Winter S (2009). Expression of organic cation transporters OCT1 (SLC22A1) and OCT3 (SLC22A3) is affected by genetic factors and cholestasis in human liver. *Hepatology*.

[31] Lawson TA, Pound AW (1974). The different susceptibility of rat liver lobes to carbon tetrachloride and dimethylnitrosamine. *British Journal of Experimental Pathology*.

[32] Aller M, Arias J, García-Domínguez J, Arias J, Durán M, Arias J (2008). Experimental obstructive cholestasis: the wound-like inflammatory liver response. *Fibrogenesis & Tissue Repair*.

[33] Gressner AM, Weiskirchen R (2006). Modern pathogenetic concepts of liver fibrosis suggest stellate cells and TGF-*β* as major players and therapeutic targets. *Journal of Cellular and Molecular Medicine*.

[34] Friedman SL (2008). Mechanisms of hepatic fibrogenesis. *Gastroenterology*.

[35] Parola M, Marra F, Pinzani M (2008). Myofibroblast-like cells and liver fibrogenesis: emerging concepts in a rapidly moving scenario. *Molecular Aspects of Medicine*.

[36] Weerachayaphorn J, Luo Y, Mennone A, Soroka CJ, Harry K, Boyer JL (2014). Deleterious effect of oltipraz on extrahepatic cholestasis in bile duct-ligated mice. *Journal of Hepatology*.

[37] Yang M, Ramachandran A, Yan H (2014). Osteopontin is an initial mediator of inflammation and liver injury during obstructive cholestasis after bile duct ligation in mice. *Toxicology Letters*.

[38] Matsuzaki S, Onda M, Tajiri T, Kim DY (1997). Hepatic lobar differences in progression of chronic liver disease: correlation of asialoglycoprotein scintigraphy and hepatic functional reserve. *Hepatology*.

[39] Jacobsson H, Jonas E, Hellström PM, Larsson SA (2005). Different concentrations of various radiopharmaceuticals in the two main liver lobes: a preliminary study in clinical patients. *Journal of Gastroenterology*.

[40] Larson SP, Bowers SP, Palekar NA, Ward JA, Pulcini JP, Harrison SA (2007). Histopathologic variability between the right and left lobes of the liver in morbidly obese patients undergoing Roux-en-Y bypass. *Clinical Gastroenterology and Hepatology*.

[41] Sumiyoshi T, Shima Y, Tokorodani R (2013). CT/99mTc-GSA SPECT fusion images demonstrate functional differences between the liver lobes. *World Journal of Gastroenterology*.

